# Integrated Genetic Diversity and Multi-Omics Analysis of Colour Formation in Safflower

**DOI:** 10.3390/ijms26020647

**Published:** 2025-01-14

**Authors:** Yonghua Qin, Kangjun Fan, Aidiya Yimamu, Peng Zhan, Lu Lv, Gang Li, Jiao Liu, Zunhong Hu, Xingchu Yan, Xueli Hu, Hong Liu, Rui Qin

**Affiliations:** 1 Hubei Provincial Key Laboratory for Protection and Application of Special Plant Germplasm in Wuling Area of China, College of Life Sciences, South-Central Min Zu University, Wuhan 430074, China; 2 Industrial Crop Research Institute of Yunnan Academy of Agricultural Sciences, Kunming 650205, China; 3 Oil Crops Research Institute (OCRI) of the Chinese Academy of Agricultural Sciences (CAAS), Wuhan 430074, China

**Keywords:** *Carthamus tinctorius* L., genetic diversity, core germplasm, transcriptome, metabolome

## Abstract

Safflower (*Carthamus tinctorius* L.) is a medicinal and edible cash crop that is widely cultivated worldwide. However, the genetic diversity of safflower germplasm resources and the reasons for the variations in safflower flower colour remain unclear. In this study, we used a combination of agronomic traits and Indel markers to assess the genetic diversity of 614 safflower germplasm resources. The results showed that most of the evaluated agronomic traits had high variability. The mean values of the Shannon’s information index (I) and polymorphism information content (PIC) in 50 pairs of Indel markers were 0.551 and 0.296, respectively. The population structure, neighbour-joining phylogeny, and principal coordinate analyses classified all genotypes into four subgroups, and 214 safflower core germplasms were constructed. Multiple analyses of genetic diversity parameters, range conformity, and the percentage of variance difference showed that the core germplasm did not differ significantly and could represent the original germplasm better. Transcriptome and metabolome analyses revealed that flavonoid synthesis-related genes, including *CHS*, *F3H*, *ANS*, and *BZ1*, were differentially expressed in different coloured safflowers. Most significantly, different genes and metabolite compounds in white safflowers were enriched upstream from the phenylpropanoid metabolic pathway to the production of naringenin, whereas those in red safflowers were concentrated in the downstream pathway from eriodictyol. Meanwhile, the preliminary quantification of anthocyanins and carotenoids extracted from red, orange, and white types of safflower showed that the level of both anthocyanins and carotenoids were highest in red types. This work provides new insights into the formation of different safflower flower colours and in the conservation and management of safflower germplasm.

## 1. Introduction

Safflower (*Carthamus tinctorius* L.) is a flowering annual plant of the Asteraceae family that originated in the Fertile Crescent between southern Israel and western Iraq, where it was first cultivated about 4000 years ago [1]. Vavilov suggested that the three centres of origin of safflower are India, Afghanistan, and Ethiopia [2]. According to the Food and Agriculture Organization of the United Nations (FAO), the global cultivation area of safflower is 1,204,335 hectares, with a production of 1,002,023.32 tons. The major production areas for safflower seeds are Asia and the Americas, accounting for 90% of cumulative production [3]. In China, safflower was introduced to the Central Plains more than 2000 years ago during Zhang Qian’s mission to the West during the Han Dynasty. Currently, Xinjiang, Yunnan, Sichuan, and Gansu provinces are the major production areas, with a cultivation area of 23,069 ha and production of 33,879.09 tons [4]. Safflower is a multipurpose crop that was originally grown as a natural dye and food flavouring [5]. Since the first half of the 20th century, it has been grown as an oilseed crop, and its seeds are rich in unsaturated fatty acids (oleic, linoleic, and linolenic acids), which is directly linked to cardio-protective, hypolipidemic, anti-atherosclerotic, and anti-inflammatory effects [6]. The planting of safflower is extended to other regions worldwide given the gradual attention paid to its efficacy. However, its cultivation is affected by factors such as the yield, low oil content, susceptibility to a variety of biotic stresses, and the presence of thorns [7]. Therefore, the conservation and sustainable use of genetic resources are key to the continuous improvement of safflower.

Genetic diversity is the foundation of biodiversity and the driving force behind the stability and continued evolution of species [8]. With the development of science and technology, molecular marker technology has become one of the main ways to study genetic diversity, population structure, and relatedness. The limited genomic data of safflower compared with its major crops limit the use of applicable molecular markers. Randomly amplified polymorphic DNA (RAPD) [9], amplified fragment length polymorphisms (AFLPs) [10], simple sequence repeats (SSRs) [11], and sequence-related amplified polymorphism (SRAP) [12] are used to identify and evaluate safflower varietal resources. However, these molecular markers can only obtain limited genetic information in populations. With the release of a high-quality safflower genome [13], the insertion/deletion (Indel) marker has been developed with 11 oil traits to construct the safflower core germplasm [6]. This Indel marker has easy to design primers, basic PCR systems, and agarose gel electrophoresis for visualisation [14]. Therefore, the construction of the representative core germplasm by combining Indel markers with agronomic trait data provides a means to increase the degree of variation and genetic diversity of the core collection.

As a visible feature of plants, colour is important for their growth and development. The flower colour depends on the plant pigments such as flavonoids, carotenoids, and betalains [15]. Anthocyanins are water-soluble pigments belonging to the flavonoid family and contribute mainly to red and blue colours. Carotenoids are the natural pigments that impart yellow, red, or orange colours to flowers, and most plants have similar contents, including β, ε-carotenoids and β, β-carotenoids in their green tissues. For example, deep red or purple chrysanthemums have high levels of anthocyanins, while deep yellow or green chrysanthemums have high amounts of carotenoids [16]. In general, flavonoid biosynthetic pathways begin with phenylacetone biosynthesis, and chalcone synthase produces the chalcone scaffold from which all flavonoids are derived. The quinoxaline chalcone C-glycoside is one of the chalcone scaffolds present only in safflower species. Almost all red and yellow pigments in safflower petals belong to the C-glycoside quinoxalinone family of flavonoids. Hydroxysafflor yellow A (HSYA) is the main component of the flavonoids in safflower and consists of a C-glucosyl quinoxalinone. C-glucosyl quinuclidin chalcone is formed by the glycosylation of naringenin chalcone by Cytokinin-O-Glucosyltransferase (CGT), followed by oxidation by cytochrome P450 (P450). In addition, a high-performance liquid chromatography (HPLC) analysis of yellow pigments in the aqueous extracts of safflower found that the predominant constituent was HSYA, followed by anhydrosafflor yellow B (AHSYB) [17]. Although the biosynthetic pathways of flavonoids and carotenoids in other plants are well-established, the balance of the expression dynamics of the relevant genes during safflower colour transition is still poorly understood.

Changes in flower colour are comprehensively regulated by physiological changes, metabolite accumulation, and fluctuations in the transcript levels of relevant genes [18]. Combined metabolite–transcriptome analyses can elucidate the interactions between gene expression and metabolite accumulation, providing insights into the regulatory mechanisms [19]. So far, the integration of genomics, transcriptomics, and metabolomics has emerged a powerful tool to understand the complex network controlling changes in plant flower colour [20]. It has been widely applied to many plants such as tomato [21], watermelon [22], wolfberry [23], *Iris sanguinea* [24], *Lagerstroemia indica* [25], and other plants. Therefore, combining the advantages of multi-omics analysis can provide a more comprehensive understanding of plant variation in flower colour, which ultimately contributes to the improvement of crop traits.

Based on research findings from other plants, we hypothesise that the variation in flower colour of safflower may be related to the ratio of anthocyanin and carotenoid content. To verify this hypothesis, we developed Indel markers using safflower whole-genome and RNA sequence data, analysed the genetic diversity of 614 safflower germplasm resources collected, and constructed core germplasms. Transcriptome and metabolome analyses were also used to resolve significantly differentially accumulated metabolites and significantly different genes in three different colours of safflower. This work provides a basis for the research on the dynamic change of metabolite accumulation and the expression of the key genes involved in safflower colour formation.

## 2. Results

### 2.1. Genetic Diversity Analysis of Safflower Germplasm

**Figure 1:**
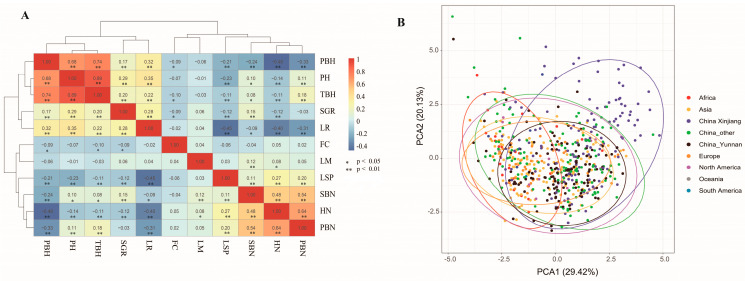
Agronomic traits analysis of 614 safflower germplasm. (**A**): Correlation analysis of 11 agronomic traits. (**B**): Principal component analysis of 11 agronomic traits.

An analysis of variance, correlation analysis, and principal component analysis were performed on 614 safflower germplasm. The results, in combination with agronomic traits data, were used to evaluate the population structure of safflower populations and effectively screen the specific germplasm. Differences were observed in seven morpho-quantitative traits of the safflower germplasm with a variation range of 29.61–77.35% (Table S1). Hence, the studied safflower germplasm is rich in genetic diversity. Various degrees of correlation were observed among the agronomic traits, with most traits exhibiting significant or highly significant correlations with each other. Plant height showed a positive correlation with height of the top branch (r = 0.89 **) and height of primary branches (r = 0.68 **). The height of primary branches showed a negative correlation with the number of heads per plant (r = −0.49 **) and a positive correlation with the height of the top branch (r = 0.74 **) (Figure 1A). The results of the principal component analysis (PCA) of the 11 traits of the 615 accessions indicated that the first four principal components explained 70.34% of the total variation (Table S2). The PCA was performed by combined PCA1 and PCA2 with geographic distribution (Figure 1B). The results demonstrated no obvious pattern in the distribution of different germplasm resources and a large distribution range, which confirmed the extensive genetic diversity in 614 safflower germplasm resources.

**Figure 2:**
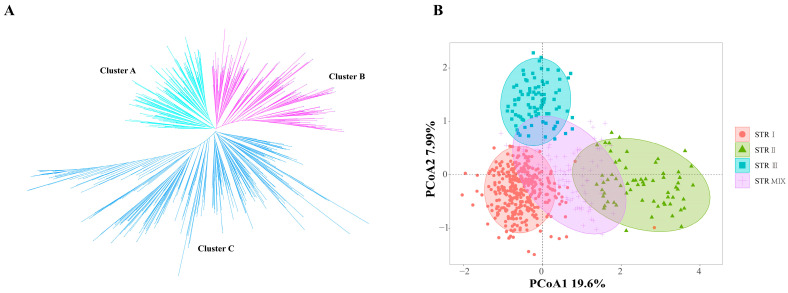
Population analysis of 614 safflower germplasm. (**A**): Cluster analysis based on neighbour-joining (NJ) method. (**B**): Score plot generated using PCoA.

A polymorphism analysis was performed on 50 pairs of Indel markers to verify the polymorphism of the developed Indel markers (Table S3). The mean value of the Shannon’s information index (*I*) was 0.551, and the polymorphism information content (*PIC*) ranged from 0.107 to 0.375, with a mean value of 0.296. An unrooted phylogenetic tree was constructed by using the neighbour-joining method with Indel markers, and it showed that 614 safflower germplasms were classified into three clades (Figure 2A). The maximum ΔK value was found when K = 3 (Figure S1A,B), suggesting that the 614 safflower accessions could be divided into three subgroups (STR I, STR II, STR III). Based on the Q-matrix and maximum membership coefficient, 87 safflower accessions had Q values less than 0.6, and the subgroup was STR MIX. According to the results of the AMOVA analysis, the inter-subpopulation variation in safflower accounted for 23.50% of the total variation, and most of the variation existed within the subpopulations (Table S4). Fst ranged from 0.069 to 0.425, indicating great genetic differentiation between STR I and STR III, and between STR II and STR III (Table S5). The principal coordinate analysis (PCoA) was carried out on 614 safflower varieties, and its results showed that four subgroups could be distinguished (Figure 2B).

Phenotypic data and genotypic data of safflower were screened using Core Hunter version 3 software. Two subsets, namely, CtCore 1 and CtCore 2, were generated (Table S6), and 214 core germplasms of safflower were obtained by combining the CtCore 1 and CtCore 2 subsets and removing the overlap. The evaluation using Indel markers demonstrated that the 214 safflower core germplasms are more representative than that of the 614 safflower germplasms (Table S7). The mean difference (MD) percentages of the core collections were less than 20%, and the range coincidence rates (CRs) were greater than 80%, indicating that all core collections met the conditions. The mean difference rate, variance difference rate (VD), range coincidence rate, and coefficient of variation coincidence rate (VR) were 0.00%, 57.14%, 98.14%, and 110.92%, respectively (Table S8). The results of the comparison of the distribution of the core germplasm in the original germplasm based on agronomic traits and Indel markers, respectively, revealed that the core subsets formed by CtCore 1 and CtCore 2 were more evenly distributed in both cluster methods (Figure S2). Therefore, the constructed core germplasm can effectively represent the population diversity of the original materials in the evaluation of agronomic traits.

### 2.2. Correlation Analysis Between Phenotype and Indel Makers

**Figure 3:**
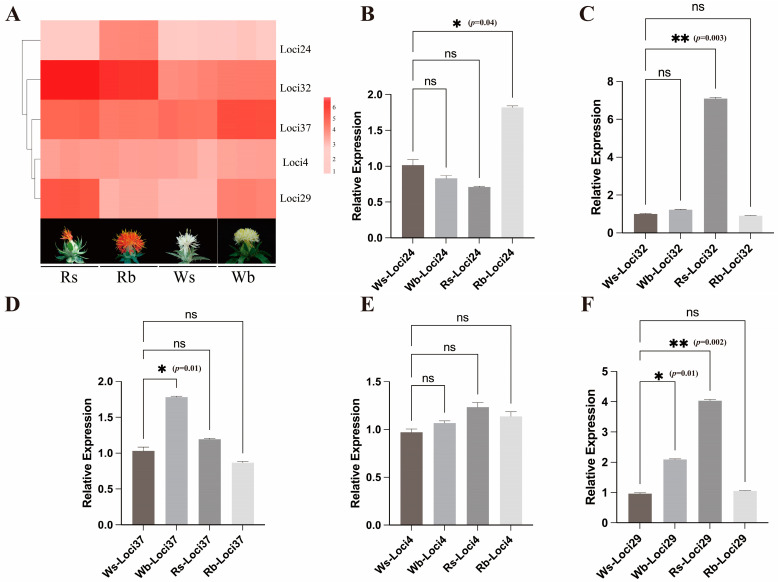
qPCR validation of phenotypic and Indel marker association analysis loci. (**A**): Heatmap of expression of five loci in red and white flower safflower at four periods. (**B**–**F**): qPCR validation of five loci, respectively. The error bars represent S.E.M. Statistically significant differences were tested by One-way ANOVA. All the above experiments were repeated three times independently.

The association analysis of safflower agronomic traits and Indel makers was performed using TASSEL version 5.0. A total of 16 loci were associated with PH, HN, PBN, PBH, TBH, SBN, and FC at *p* < 0.05. The cis-acting element analysis of the promoter region revealed that Loci4, 24, 29, 32, and 37 were associated with FC (Table S9). Loci37 was the MYB binding site for the flavonoid metabolism regulatory genes. MYB transcription factors are widely involved in the synthesis of flavonoids, and the flavonoid metabolism pathway is the main regulatory pathway of safflower flower colour change. Thus, transcriptome and qRT-PCR analyses were performed on the genes where these five loci are located (Figure 3). Loci24 is relatively highly expressed at Rb, and the gene at this locus may be involved in regulating the growth of safflower buds. Loci32 is relatively highly expressed at Rs and Rb and may be involved in the formation and growth of safflower filaments and buds. Loci37 has a MYB binding site and is involved in the regulatory pathway of safflower flavonoid metabolism by binding to MYB transcription factors; it is relatively highly expressed at Wb. Loci4 is expressed at the same level in Rs, Rb, Ws, and Wb, and may be involved in the formation of flower colour through other transcriptional regulatory pathways. Loci29 is mainly involved in the formation of small flower buds in safflower and in the regulation of large flower buds in white flowers.

### 2.3. Transcriptome and Metabolome Analyses to Compare Different Colours of Safflower

To study the physiological mechanism of different flower colour changes in safflower, we performed widely targeted metabolic profiling on samples of three different colours, namely, red (R), yellow (Y), and white (W), of safflower (Figure S3). The variation in the metabolite composition of the three different colours of safflower was assessed using LC-MS/MS, and a total of 627 compounds were detected, such as flavonoids, pyridines and derivatives, carboxylic acids and derivatives, terpenoids, and organic acids. All the metabolic species were annotated through KEGG (HSYA are special metabolic species of safflower that were not annotated by KEGG). The PCA of the samples revealed a distinct dispersion between groups and tight aggregation within groups, indicating that the sampling results were stable and reproducible (Figure S4).

**Figure 4:**
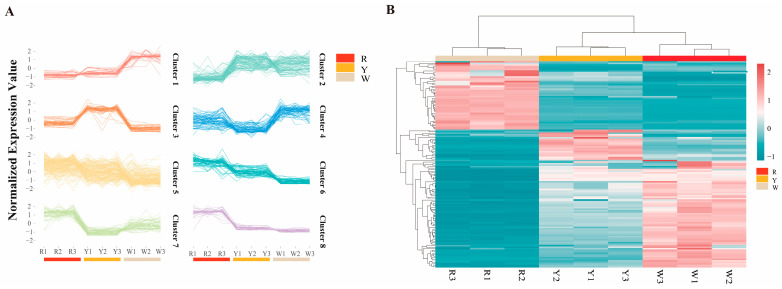
Metabolome mapping of three different flower colours. (**A**): The K means analysis of metabolites identified in three different colour groups. (**B**): Heat map of expressions levels for the Cluster1, 3, and 6 of DAMs.

The significant DAMs between pairwise comparisons were screened based on the variable’s importance in projection (VIP) ≥ 1 and *p*-value < 0.05, which indicated that 88, 96, and 83 DAMs were detected in the three comparison groups (R vs. W, R vs. Y, and W vs. Y) (Table S10). The scores plot of the OPLS-DA model discriminated the flower colours, with different colour groups all exhibiting satisfactory separation, as reflected by all samples having a significantly different metabolite composition (Figure S5A–C). The loading plot in the scores plot of the OPLS-DA model with different colour groups shows that HSYA, oxazepam glucuronide, quercetin 3-(6″-malonyl-glucoside), etc., are dominant metabolites in the R group compared with the W group. In parallel, astragalin, luteolin 7-galactoside, and kaempferol 3-alpha-D-galactoside are dominant metabolites in the W group (Figure S5D). Based on the comparison of the R group and Y groups, riboflavin, HSYA, and quercetin-3-(6″-malonyl)-glucoside are also the main metabolites in the R group, and luteolin 7-galactoside, isoquercitrin, and alpha-curcumene are the main metabolites in the Y group (Figure S5E). Kaempferol 3-alpha-D-galactoside, astragalin, and luteolin 7-galactoside are the dominant metabolites in the W group compared with that in the Y group; biorobin, datiscin, quercetin-3,4′-O-di-beta-glucopyranoside, etc., are the dominant metabolites in the Y group (Figure S5F). Most of the DAMs were enriched to the flavonoid biosynthesis (map00941), such as astragalin, luteolin 7-galactoside, isoquercitrin, cyanidin 3-glucoside, HSYA, etc. (Table 1). The k-means of DAMs were studied to investigate trends in the relative content of metabolites in different colour groups. The metabolites were well-distinguished at k = 8. Metabolites in Cluster1 were significantly higher in the W group than in the R and Y groups, indicating that these metabolites, mainly including kaempferol, quercetin, coumarin, etc., were dominant in white safflower (Figure 4A). The content of metabolites in Cluster3 was significantly higher in group Y than in the two remaining groups, and they were the major metabolites of yellow safflower (mainly including isorhamnetin, petunia pigments, herbaceous pigments, etc.). The dominant metabolites of the R group are in Cluster6 and include HSYA, lignans, catechin, etc. The hierarchical cluster analysis of all differentially expressed metabolites in Cluster1, Cluster3, and Cluster6 showed that many metabolites were highly expressed in the R and W groups (Figure 4B).

**Figure 5:**
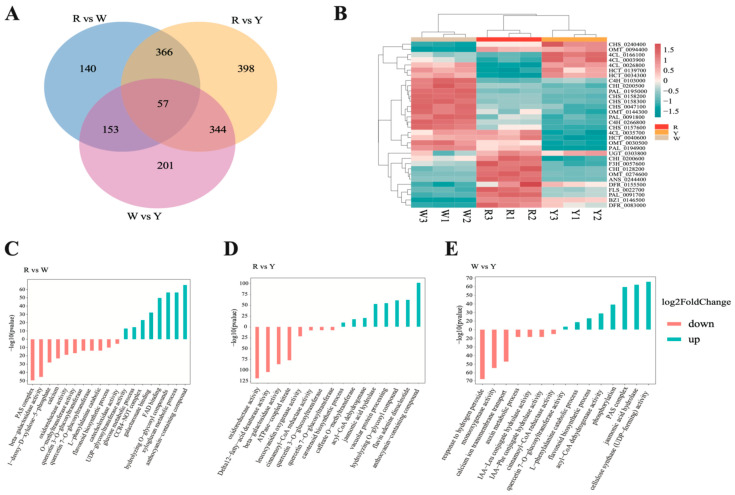
Transcriptome group mapping of three different flower colours. (**A**): Venn plots of three compared combinations with R vs. W, R vs. Y, and W vs. Y. (**B**): Expression profiles heatmap of flavonoid pathway structural genes in different colours of safflowers. (**C**): GO enrichment histogram of R group compared with W group. (**D**): GO enrichment histogram of R group compared with Y group. (**E**): GO enrichment histogram of W group compared with Y group.

Applying filtering criteria of |log2Fold Change| ≥ 1 and FDR < 0.05, a total of 2636 DEGs were detected in the three compared combinations of R vs. W, R vs. Y, and W vs. Y; 716, 1165, and 755 DEGs were identified in the compared combinations (Figure 5A). The annotation of the differential genes using the gene ontology (GO) resource revealed that they are involved in a variety of functions and pathways. The anthocyanin-containing compound biosynthetic process (GO:0009718), glucose metabolic process (GO:0006006), UDP-glycosyltransferase activity (GO:0008194), and xyloglucan metabolic process (GO:0010411) were upregulated (GO:0010411) in the R vs. W compared combination; beta-galactosidase activity (GO:0004565), quercetin 7-O-glucosyltransferase activity (GO:0080044), the L-phenylalanine catabolic process (GO:0006559), and flavonoid biosynthetic process (GO:0009813) were downregulated (Figure 5C). In the R vs. Y compared combination, the anthocyanin-containing compound biosynthetic process (GO:0009718), jasmonic acid hydrolase (GO:0120091), and carotenoid biosynthetic process (GO:0016117) were upregulated, and Delta12-fatty-acid desaturase activity (GO:0102985), beta-galactosidase activity (GO:0004565), leucocyanidin oxygenase activity (GO:0050589), and quercetin 7-O-glucosyltransferase activity (GO:0080044) were downregulated (Figure 5D). In the R colour group combinations (R vs. W and R vs. Y), the anthocyanin-containing compound biosynthetic process was enriched, and quercetin 7-O-glucosyltransferase activity and beta-galactosidase activity were enriched in both the Y and W groups. Meanwhile, jasmonic acid hydrolase (GO:0120091), cellulose synthase (UDP-forming) activity (GO:0016760), the flavonoid biosynthetic process (GO:0009813) and quercetin 7-O-glucosyltransferase activity (GO:0080044) were upregulated in the W vs. Y compared combination. The downregulated GO items included the auxin metabolic process (GO:0009850), response to hydrogen peroxide (GO:0042542), monooxygenase activity (GO:0004497), cinnamoyl-CoA reductase activity (GO:0016621), etc. (Figure 5E). Therefore, the expression levels of key enzyme genes in the flavonoid biosynthesis pathway were investigated. The expression of most upstream key enzyme genes, such as CHS, phenylalanine aminotransferase (PAL), and 4-coumarate-CoA ligase (4CL), was higher in groups W and Y than in the R group; meanwhile, the expression of downstream key enzyme genes, such as F3H, ANS, and BZ1, was higher than that of the R group (Figure 5B).

**Figure 6:**
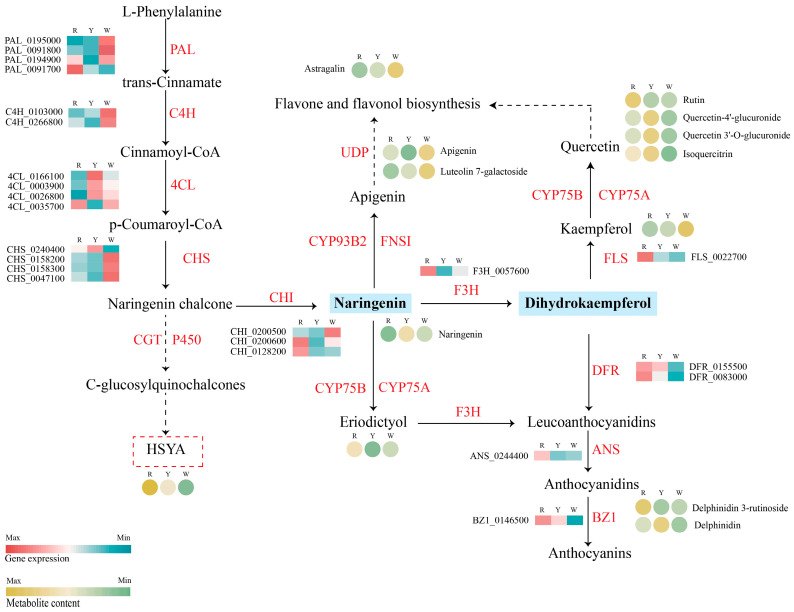
Schematic representation of structural gene expression levels and metabolite content in the biosynthetic pathway of flavonoids in different coloured safflower.

The metabolites of the flavonoid biosynthesis pathway in different coloured safflowers varied considerably due to the absence of HSYA in the W group and the different levels of HSYA in the R and Y groups. The metabolic species and the gene expression levels were integrated into a schematic diagram (Figure 6). In this pathway, L-phenylalanine is converted into p-coumaroyl-CoA by phenylalanine ammonia lyase (PAL), cinnamate-4-hydroxylase (C4H), and 4-coumarate CoA ligase (4CL); this process is common to many secondary metabolism pathways. Then, chalcone synthase (CHS) produced chalcones such as naringenin chalcone and C-glucosyl quinochalcones. The pathway of HSYA converted from C-glucosyl quinochalcones is still not well-understood. Key enzyme genes from L-phenylalanine to dihydrokaempferol have higher expression levels in the W and Y groups than in the R group; from dihydrokaempferol, most enzymes in the anthocyanin metabolic pathway such as ANS, FLS, and BZ1, were highly expressed in the R group. The comparison of the R and Y groups showed a distraction beginning downstream from quercetin. Isoquercitrin, quercetin, and glucuronide were higher in the Y group, and rutin was high in the R group. Those flavonoids from naringenin in the R group and W group show different flow directions. In the W group, apigenin and kaempferol flowed more, whereas in the R group, the anthocyanin metabolic pathway flowed more. Apart from that, the W and Y group safflower flavonoids were different from dihydrokaempferol.

**Figure 7:**
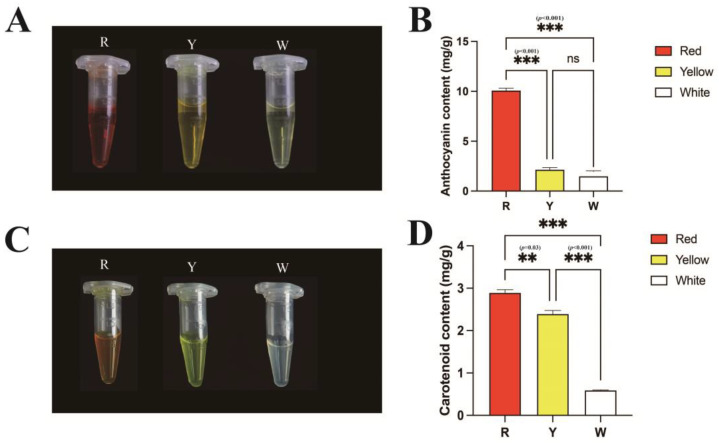
Two pigments were extracted from the different coloured flowers of safflower. (**A**,**B**): Three types of floral anthocyanin extracts and contents. (**C**,**D**): Three types of floral carotenoids extracts and contents. The error bars represent S.E.M. Statistically significant differences were tested by One-way ANOVA. All the above experiments were repeated three times independently.

The content of safflower yellow (SY) in safflower is about 20–30%, and HYSA is one of the constituent compounds, which account for 80–90% of the total SY. To investigate the other pigments in safflower, we extracted anthocyanin and carotenoid from three different colours of safflowers, R, Y, and W. The contents were initially determined by the spectrophotometric method. The anthocyanin extracts showed different colours among the three colours groups; the R group had deep-red colours, the Y group had yellow colours, and the W group had less-yellow colours (Figure 7A). The absorbance of these extracts was recorded at 520 nm and quantified in each colour group. In comparison with the other groups, the anthocyanin content was higher in the R group, and had an average value of 10 mg/g. The anthocyanin content was very low in groups Y and W (Figure 7B). The extraction of carotenoids from the three colour groups showed that the Y group is yellow in colour and the W group is nearly transparent. The absorbance of carotenoids was recorded at 440 nm. Compared with that in Y group, the total carotenoid content was higher in the R group and lower in the W group (Figure 7C,D).

## 3. Discussion

Agronomic traits are unique to crop varieties and are determined by their genetic background and environmental influences. In this study, we analysed the agronomic traits of 614 safflower varieties. The coefficients of variation of seven quantitative agronomic traits were greater than 25%, indicating significant genetic variation in the population. The correlation results indicated that the agronomic traits of safflower may have mutual constraints and influences and therefore should be considered and analysed comprehensively during germplasm creation. The PCA showed that the distribution trends of the safflower germplasm in China and abroad differed somewhat, indicating that the size of the range of the selected area and the intricacies of the environment should be fully considered. Meanwhile, the population structure analysis, phylogenetic analysis, and PCoA results showed that the collection of 614 safflower germplasms was assigned to four subgroups. Kumar et al. worked on 135 different safflower genotypes with SRAP and SSR markers, which showed four clusters within the safflower germplasm, while there was no strong correlation between geographic origin and estimated genetic diversity [11]. Based on AMOVA, the genetic diversity within populations was more pronounced than between populations, and most of the genetic variation was present within populations. This finding is consistent with other results [12].These results indicate that the 614 safflower germplasm is highly differentiated and rich in genetic diversity, which provides valuable material for molecular breeding.

The core germplasm can represent the genetic diversity level of the original population to the greatest extent by selecting a small, representative genetic resource from the whole, and can maximise the genetic diversity of the germplasm in a limited breeding cycle [26]. This is because agronomic trait data can be used to visualise plant differences and are easy to manipulate. However, agronomic traits are greatly influenced by the environment, especially for the germplasm collected from different regions [27]. Therefore, core germplasm populations are usually constructed based on molecular data, supplemented by agronomic trait data. The exploitation of the genetic diversity of genetic resources in safflower is important to construct the core germplasm and for improving breeding studies. In this paper, 214 core germplasm sources of safflower were constructed by combined agronomic traits and Indel markers using Core Hunter 3. The results showed high levels of dependence for most parameters, indicating that the selected core germplasm maintained the genetic diversity of the native germplasm. The established safflower core collection can be used in future genome-wide association studies. It can also play an important role in diversifying the genetic base of the working collection as well as in breeding programs. Therefore, the construction method has been validated in species such as *Robinia pseudoacacia* L. and *Juglans regia* L. [28,29].

In recent years, the application of association analysis in plant molecular marker-assisted breeding has effectively accelerated the selection of superior varieties. Especially in crops, 91 significant trait-related SNPs were detected by marker-trait association analysis using GLM, MLM, and mrMLM in cotton; among them, 33 were related to PSB, 21 were related to SB, and 37 were related to AB [30]. A total of 96 loci were identified for eight agronomic traits using GLM and MLM in safflower [31]. Transcriptomic and qRT-PCR analyses of the genes for five loci (Loci4, Loci24, Loci29, Loci32 and Loci37) in the association analyses showed that they were associated with the formation of flower colour, indicating that the phenotypic identification of the Indel marker screen was consistent. Based on these results, the reliability of the marker-phenotype association was demonstrated, and the applicability of the core germplasm constructed on the basis of Indel markers was illustrated. However, it is not sufficient to identify putative candidate genes, which may be involved in complex biological pathways, because there is still a lot of information missing. Fully understanding these metabolic pathways could help to elucidate the precise role of these genes affecting particular traits and could be a good starting point to obtain high-quality safflower. Thus, additional studies, such as QTL mapping and functional validation by means of molecular cloning, will be required in the future.

Flower colour is the result of the synergistic accumulation of several metabolites, especially flavonoids and carotenoids. The expression levels of structural genes were further examined in this study. Based on the transcriptome results, the anthocyanin biosynthetic process and most metabolic processes associated with glycosylation were enriched in the R group, indicating that anthocyanins are generally found in the form of glycosides. The flavonoid biosynthetic process, L-phenylalanine catabolic process, and catechol oxidase activity were enriched in the W group, which indicates that more flavonoid compounds are accumulated in white flowers. On the significant DEGs, most of the upstream expressed genes such as *4CL*, *CHI*, and *CHS* have higher expression levels in the W and Y groups compared with the R group, and the majority of enzymes in the anthocyanin metabolic pathway, such as *ANS*, *FLS*, and *BZ1*, were highly expressed in the R group. The expression of these genes is increased and the metabolites are increased through catalysis by various enzymes, which ultimately leads to the formation of more colourless anthocyanins, which were catalysed into coloured anthocyanins by the dehydrogenation, isomerization, and dehydration of the ANS genes under acidic conditions [32]. This probably a consequence of different types of flavonoid compounds like flavones, flavanols, and anthocyanins. Differences in accumulated levels were observed among the three colour groups of safflowers; anthocyanins were accumulated in the R group, whereas anthocyanins were lacking in white flower, and more colourless flavonoids like apigenin, kaempferol, and naringenin were accumulated in the W group. These findings are supported by previous studies that have emphasized the importance of transcriptome analysis in understanding flavonoid biosynthesis. For example, Hong et al. used the mechanism of the effect of colour change on *CtbHLH41*, *CtMYB63*, and *CtWD40-6* of Sichuan safflower to demonstrate that *CtMYB63/CtWD40-6* enhances the transcriptional activity of *CtbHLH41* on *CtDFR* (dihydroflavonol 4-reductase) to accumulate anthocyanins [33]. Hong et al. found that the differential expression of *F3′5′H* and F3′H is a factor in the colour diversity of crape myrtle [25].

Based on the metabolome results, naringenin chalcone is catalysed by a series of enzymes to produce naringenin, and the W group safflower metabolites downstream of naringenin begin to differ from those of the R group and Y group safflowers. The metabolites of the W and Y groups of safflowers flowed more to dihydrokaempferol and downstream of kaempferol and quercetin. Metabolites in the R group safflower flowed more to eriodictyol and anthocyanidins. It is the different metabolic flows in naringenin and eriodictyol that lead to the different contents of delphinidin and downstream anthocyanin-related products, which may be the main reason for the formation of different flower colours. These findings are supported by previous studies, in which Wang et al., using transcriptomic and metabolomic analyses, found that differential expression of the *CHS* gene was one of the main reasons for the differences in flavonoid species and content between different coloured safflowers [34]. Further, the initial quantification of anthocyanin and carotenoid contents in different coloured safflower flowers (red, yellow, and white) with extracted anthocyanins and carotenoids showed that the differences in safflower flower colours were not only related to flavonoid chemistry, but also co-determined the flower colours together with several other pigments, which led to a rich variety of colours in the flowers. These findings provide a wealth of data for future use in genome-wide association analysis.

## 4. Materials and Methods

### 4.1. Plant Materials and Samples Collection

A total of 614 safflower materials were provided by the germplasm repository of the Wuhan Institute of Oilseed Crops, Chinese Academy of Agricultural Sciences. The safflower material was planted in an experimental field at 58 m above sea level in Huangpi District, Wuhan City, Hubei Province, China (30°45′59″ N, 114°27′46″ E). Each safflower variety was sown in a row 4 m long, 50 cm apart, with an average plant spacing of 40 cm. The total number of seedlings left in each row after interplanting was 10.

Agronomic traits were investigated following the guidelines provided by the Oils Crops Research Institute of the Chinese Academy of Agricultural Sciences for safflower [35] (Table S11). These guidelines encompass the following: plant height (PH), primary branch height (PBH), terminal branch height (TBH), number of primary branches (PBNs), number of secondary branches (SBNs), seed germination rate (SGR), load resistance (LR), flower colour (FC), number of heads per plant (HN), leaf margin with thorns or without thorns (LSP), and leaf margin (LM). The data were recorded for five healthy plants of each accession.

### 4.2. DNA Extraction and Indel Marker Development

The best growing plant from the five plants with good consistency was selected as the sample plant, and the leaves were collected and stored at −20 °C. Genomic DNA was extracted using a modified version of the cetyltrimethylammonium bromide (CTAB) method [36]. The quality and quantity of the extracted genomic DNA were determined by agarose gel electrophoresis and spectrophotometry. The DNA concentration was diluted to 50 ng/μL, and the diluted DNA was placed and stored at −20 °C for subsequent studies.

Indel markers were developed based on the safflower whole genome and transcriptome data [13]. The specific methods were as follows: (1) Illumina sequencing of multiple safflower accessions, (2) removal of low-quality sequences by using Trimmomatic version 0.35 software (3) alignment of processed transcriptome sequences to safflower genome sequences by utilizing Burrows–Wheeler Aligner (BWA) version 0.7.17 software, (4) use of GATK software to find insertion/deletion sites (Indel) and retain only Indel sites ≥ 20 bp in length for subsequent detection by agarose gel electrophoresis, and (5) use of Primer version 5.0 software to design primers based on flanking sequences of Indel sites with the following default parameters: primer length 18–25 bp, annealing temperature 57–63 °C, GC content 50–60%, and product length 100–300 bp (Table S12).

### 4.3. Analysis of Genetic Diversity in Safflower Germplasm

Five core parameters, including number of different alleles (*N_A_*), number of effective alleles (*N_E_*), Shannon’s coefficient, polymorphism information content (*PIC*), and expected heterozygosity (*H_e_*) were evaluated using GenAlEx version 6.51 and POPGENE version 1.3.2 software [37,38].

The Nei 1983 Distance method, neighbour-joining (NJ) tree method, and bootstrap number were selected by POWERMAKER version 3.25 and set to 1000 times to generate a marker-based tree file [39]. Based on the UPGMA algorithm on Past 3 software, the similarity coefficient was selected as Bray–Curtis, and the bootstrap number was set to 1000 times to generate a phenotype-based tree file [40]. An evolutionary tree was prepared by iTOL webtool version 5.

Core Hunter version 3.0 was employed to screen safflower core germplasms by using marker-based data and phenotypic data as input data formats [41]. Gower’s distance was utilised to calculate distances to phenotypic traits. Modified Roger’s distance and Cavalli–Sforza and Edwards distance were supported for genetic marker data. Average entry-to-nearest-entry distance was selected as the evaluation measure for core germplasm screening.

Kinship coefficients between individuals were calculated using TASSEL version 3.0 software, based on Indel marker type data [42]. The General linear model (GLM) and Mixed linear model (MLM) included in the software were used to combine the phenotypic data to perform the association analysis. In the GLM analysis, the Q value was applied as a covariate for the analysis, and the marker-phenotype association loci were identified by regression analysis using phenotype data. In the MLM analysis, the kinship K value was added as a covariate for association analysis to identify marker-phenotype association loci.

### 4.4. Metabolomic and Transcriptomic Analyses

Three different colours of safflower material, red (R), white (W), and yellow (Y), were used for metabolome and transcriptome sampling (Figure S3). When the three different coloured safflowers were in full bloom, the sampled flowers were quickly placed into a cryogenic liquid nitrogen tank for temporary storage and then transported back to the laboratory, where they were stored at −80 °C for freezing. Safflower inflorescences of the same colour were mixed to form one sample, and three replicates were taken per 100 mg. Half of each replicate was used for RNA sequencing, and the other half was used for metabolome sequencing.

The extracted samples of safflower were separated on the UHPLC system (ExionLC™ AD) equipped with Waters ACQUITY ULC HSS T3 (1.8 μm, 2.1 mm × 100 mm). The analysis conditions were as follows: column temperature, 35 °C; injection volume, 2 μL; and flow rate, 0.3 mL/min. The mobile phases were water (0.1% formic acid) (phase A) and acetonitrile (0.1% formic acid) (phase B). The gradient program of phase A/phase B was 98:2 (*v*/*v*) at 0 min, 98:2 (*v*/*v*) at 2 min, 2:98 (*v*/*v*) at 11 min, 2:98 (*v*/*v*) at 13 min, and 98:2 (*v*/*v*) at 15 min. Samples were inserted into quality control (QC) samples in queue mode to monitor and evaluate the stability of the system and the reliability of the experimental data. Mass spectrometry was carried out using the Thermo high resolution mass spectrometry (Thermo QE Focus, Shanghai, China) and data were collected using IDA (information-dependent acquisition) mode. The ion source conditions were as follows: Spray Voltage: +3500/−3500 V, Capillary Temperature: 350 °C, Sheath Gas: 30, Aux Gas: 10, CE: 10, 30, and 50. The raw data were adjusted for peak alignment, peak area extraction, retention time correction, and feature extraction using the XCMS version 3.6.3 software. MRM data were processed using Skyline version 21.1.0.146 software. The principal components analysis (PCA), orthogonal partial least squares discriminant analysis (OPLS-DA) models, and K-means clustering were used to distinguish the significant differentially accumulated metabolites (DAMs) in different colour groups.

Three groups of safflower tubular flower samples with R, W, and Y colours and biological replicates for each group were collected and analysed using transcriptome sequencing. Total RNA was isolated from frozen flowers by using a Quick RNA isolation Kit (Huayueyang, Beijing, China) according to the instructions for preparing for sequencing. The library preparations were sequenced on an ANORODA Next Seq 550AR platform and 150 bp paired-end reads were generated. The raw paired-end reads were cleaned through removing adaptor sequences, poly-N, and low-quality sequences. Clean reads after quality control were compared to the reference genome by HISAT2 to obtain position information on the reference genome or gene as well as specific sequence characteristic information of the sequenced samples [43]. The genes were quantified with FPKMs using StringTie, and a differential expression analysis of three groups was performed using the DESeq2 R package [44]. The threshold *p*-value in multiple tests to judge the significance of the gene expression difference was based on the false discovery rate (FDR) method. When FDR ≤ 0.05 and FPKM values showed at least a 2-fold difference among samples, the gene was considered a significant DEG.

### 4.5. Determination of Anthocyanin and Carotenoid Content

In order to quantify the anthocyanin and carotenoid content, the three groups of safflowers tubular flower samples with R, O, Y, and W colours were rapidly ground to powder under liquid nitrogen. Afterward, the dried samples were stored at a low temperature for subsequent experiments. The optimal conditions used to extract anthocyanins were as follows: 60% ethanol (pH = 3), soil–liquid ratio of 1:15 (g/mL), ultrasonic time of 120 min, and ultrasonic temperature of 40 °C. A potassium chloride (KCl) buffer solution with pH = 1.0 and a sodium acetate buffer solution with pH = 4.5 were used for the dilution of each extracted anthocyanin samples from the three groups of safflowers separately. The absorbance of anthocyanins was measured at 530 nm using a spectrophotometer and three replicate samples were performed for each group [45]. The anthocyanin yield rate in tubular flowers from the three group samples was calculated by the following Formulas (1) and (2):

A = (A520 nm − A700 nm)_pH1.0_ − (A520 nm − A700 nm)_pH4.5_ (1)

Anthocyanin (mg/g) = A × MW × DF × 10^3^/(ε × ℓ) (2)

A: Absorbance; ε = 4.62 × 10^6^ (Anthocyanin Extinction Coefficient); DF: Dilution factor.

Carotenoids were extracted from the three group samples using a petroleum ether–acetone solution = 5:1 (*v*/*v*) with a 40 °C-water bath for 2 h. The carotenoid absorbance was measured at 440 nm and the carotenoid content was calculated by the following Formula (3) [46]:

(3) $$\mathrm{Carotenoids}\;\mathrm{content}\;(\mu g/g)=\frac{{A\times V(mL)\times 10}^{4}}{A_{1\mathrm{cm}}^{1\%}\times P(g)}$$

*A*: Absorbance; *V*: Total extract volume; *P*: sample weight; and $A_{1\mathrm{cm}}^{1\%}$ = 2592 (β-carotene Extinction Coefficient in petroleum ether).

### 4.6. Statistical Analysis

614 safflower germplasms were used as samples, ten plants of each variety were planted, and the average agronomic traits of five plants of each variety were taken as one sample. The mean, maximum, and minimum of agronomic traits, as well as their coefficients of variation and correlation analysis (computed as Pearson’s correlation coefficient r), were analysed using SPSS 25. The formula for calculating the coefficient of variation is as follows: CV (%) = (SD/MN) × 100, where SD is the standard deviation and MN is the mean. The range is calculated as Max value–Min value. Principal component analysis (PCA) was performed using the FactoMineR and factoextra R packages [47]. F-statistics, including Fst, hierarchical AMOVA, and the pairwise Fst were conducted using GenAlEx version 6.502. GraphPad Prism version 10 was utilised for additional data visualisations and statistical analyses.

## 5. Conclusions

In this study, 214 safflower core germplasms were constructed based on 11 agronomic traits and Indel markers. The association analysis of the core germplasm population identified five loci associated with flower colour. The combined metabolomics and transcriptomics analyses showed that there are differences in flavonoid metabolic pathways and differences in anthocyanin and carotenoid contents in safflowers with different flower colours, which contribute to the richness of flower colours. These findings provide a basis for understanding the molecular mechanisms underlying safflower flower colour diversity.

## Figures and Tables

**Table 1:** Significantly different metabolites among different colours of safflower.

Metabolite Name	KEGG/Pub Chem	R vs. W	R vs. Y	Y vs. W
	Number	VIP	VIP	VIP
Astragalin	C12249	6.32	3.52	6.10
Luteolin 7-galactoside	C01514	6.26	4.71	5.48
Safflomin A	-	5.09	4.22	4.22
Oxazepam glucuronide	C07359	4.79	4.10	3.88
Quercetin-3,4′-O-di-beta-glucopyranoside	4478806	4.71	0.79	4.82
Kaempferol 3-alpha-D-glucoside	44258736	5.38	4.53	5.20
Isoquercitrin	C05623	4.16	5.07	6.01
Luteolin-3′,7-di-O-glucoside	4590322	4.58	3.17	4.16

## Data Availability

All data in this study will be available from the corresponding author upon reasonable request.

## Supplementary Materials

The following supporting information can be downloaded at: https://www.mdpi.com/article/10.3390/ijms26020647/s1.

## Abbreviations

The following abbreviations are used in this manuscript:

RAPD	Randomly amplified polymorphic DNA
SSR	Simple sequence repeats
SRAP	Sequence-related amplified polymorphism
Indel	Insertion/deletion
HSYA	Hydroxysafflor yellow A
AHSYB	Anhydrosafflor yellow B
CGT	Cytokinin-O-Glucosyltransferase
P450	Cytochrome P450
PBH	Height of primary branches
PH	Plant height
TBH	Height of the top branch
SGR	Seed germination rate
LR	Lodging resistance
FC	Flower colour
LM	Leaf margin
LSP	With or without spines on leaf margin
SBN	Number of secondary branches
HN	Number of heads per plant
PBN	Number of primary branches
PAL	Phenylalanine ammonialyase
Rs	Red-flowered small buds
Rb	Red-flowered large buds
Ws	White-flowered small buds
Wb	White-flowered large buds
MD	Mean difference percentages
CR	Range coincidence rates
VD	Variance difference rate
VR	Variation coincidence rate
C4H	Cinnamic acid 4-hydroxylase
4CL	4-coumarate-CoA ligase
CHS	Chalcone synthase
CHI	Chalcone isomerase
UDP	Uridine diphosphate
FNS I	Flavonesymhase I
F3H	Flavanone 3-hydroxylase
FLS	Flavonol synthase
DFR	Dihydroflavonol 4-reductase
ANS	Anthocyanidin synthase
BZ1	Bronze 1
