# Supplementary material for: Integrated Genetic Diversity and Multi-Omics Analysis of Colour Formation in Safflower
Source: Int J Mol Sci. 2025 Jan 14;26(2):647. doi: 10.3390/ijms26020647 (PMC11765828; doi:10.3390/ijms26020647)
Supplement: Supplementary file 1 [file ijms-26-00647-s001.zip › FigS2.pdf]

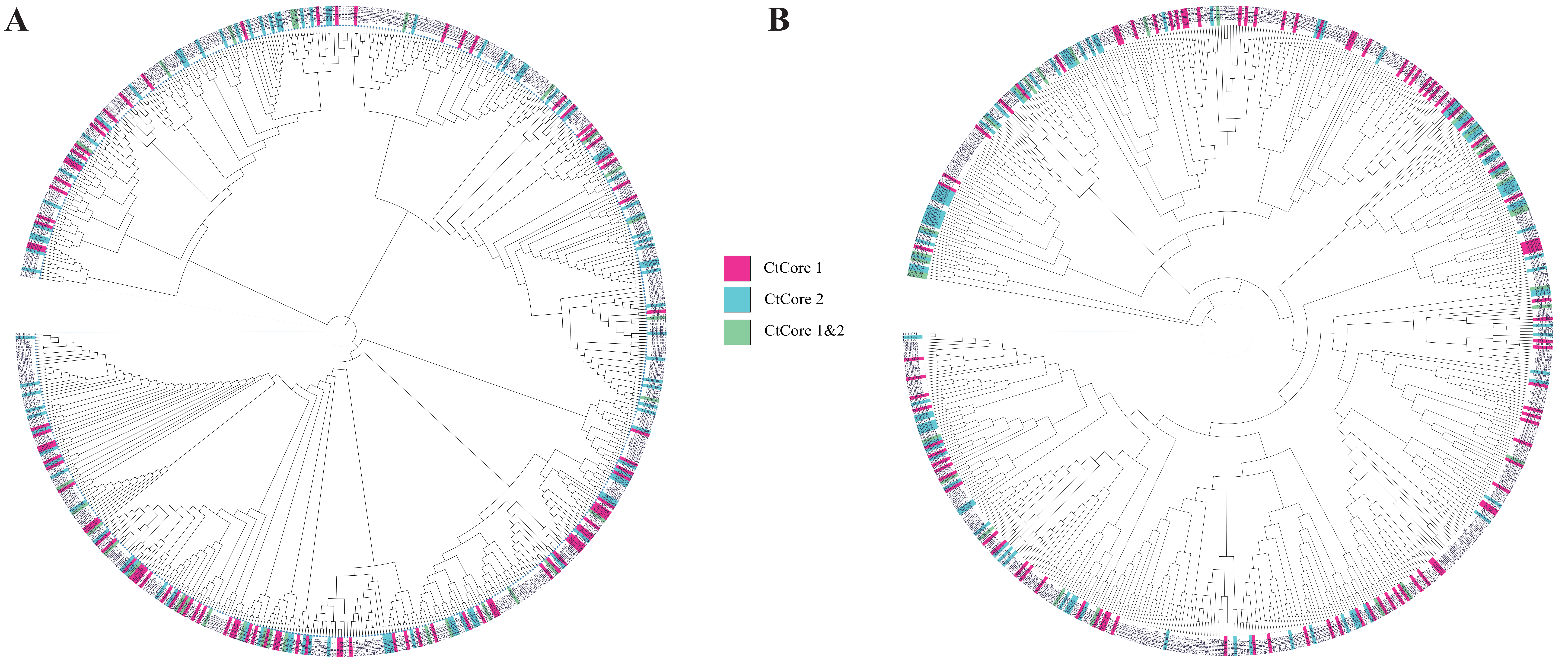
A
B
CtCore 1
CtCore 2
CtCore 1&2
