# Supplementary material for: Integrated Genetic Diversity and Multi-Omics Analysis of Colour Formation in Safflower
Source: Int J Mol Sci. 2025 Jan 14;26(2):647. doi: 10.3390/ijms26020647 (PMC11765828; doi:10.3390/ijms26020647)
Supplement: Supplementary file 1 [file ijms-26-00647-s001.zip › Supplementary Fig.pdf]

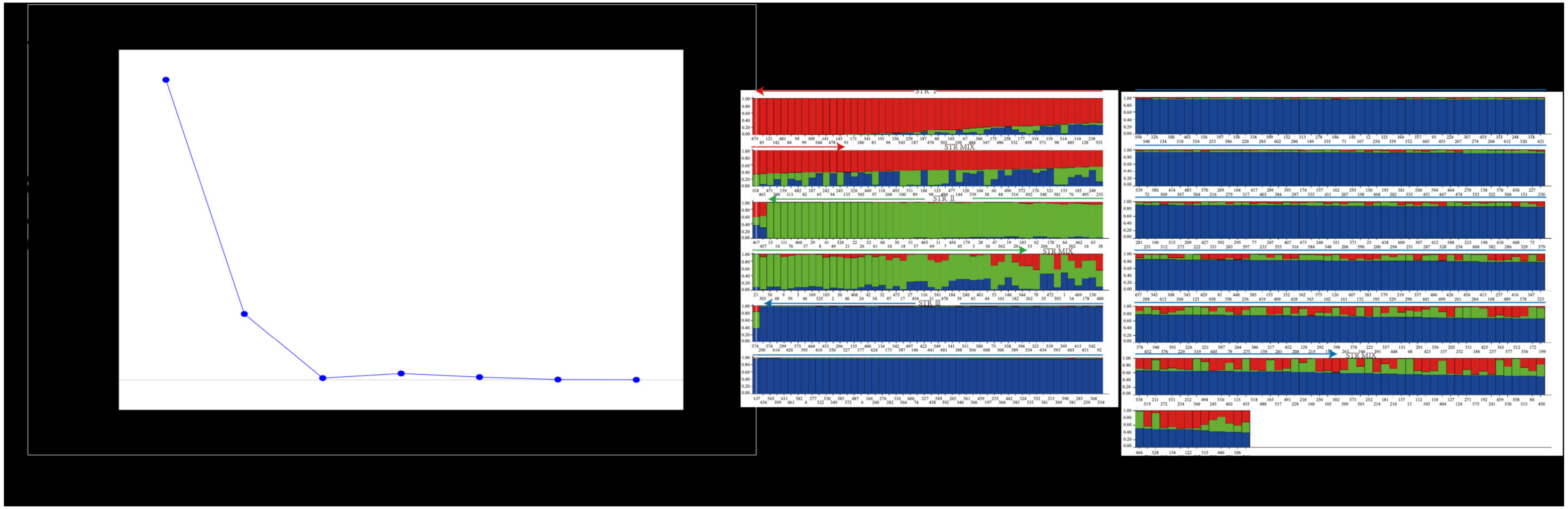

Figure S1. Population structure analysis of 614 safflower germplasm. A: Delta K that shows the population size. B: Population genetic structure based on the Bayesian clustering model with four subgroups at K = 3.

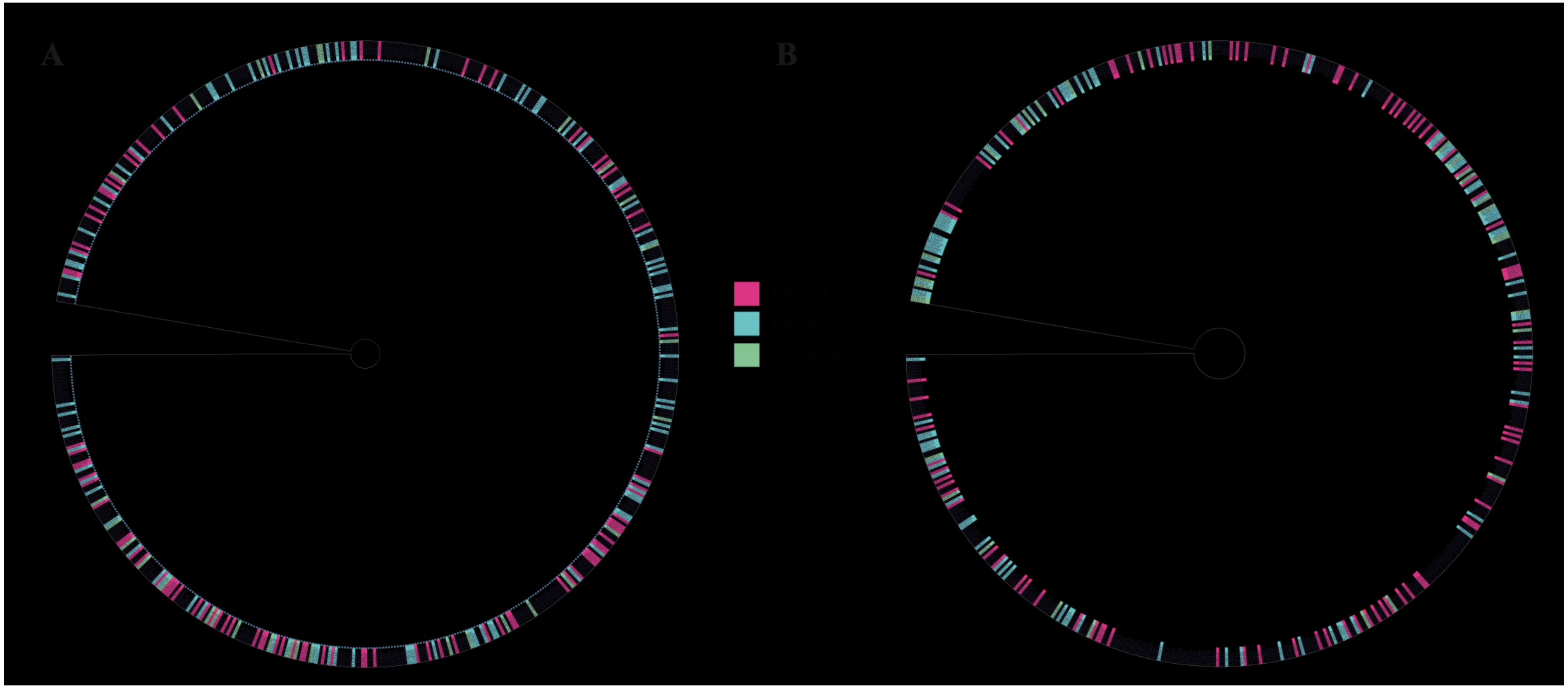

Figure S2. Distribution of core varieties in phenotypic and marker cluster maps. A: Clusters of 614 safflower germplasm were constructed based on InDel marker data and Neighbour-joining (NJ) method. B: Clusters of 614 safflower germplasm were constructed based on agronomic traits data and UPGMA method.

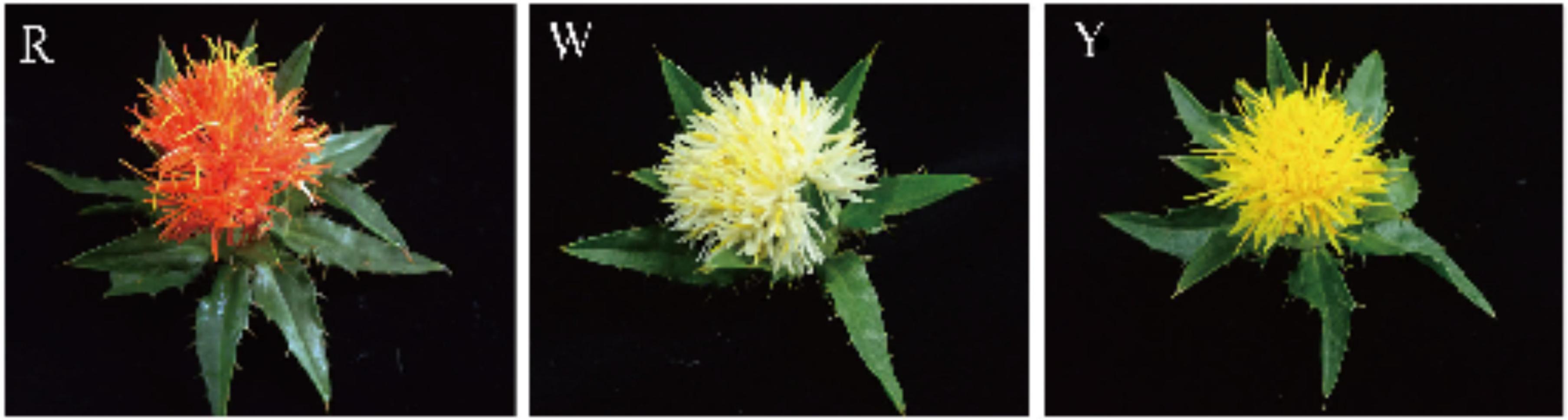

Figure S3. Plant Materials of different colours with safflowers.

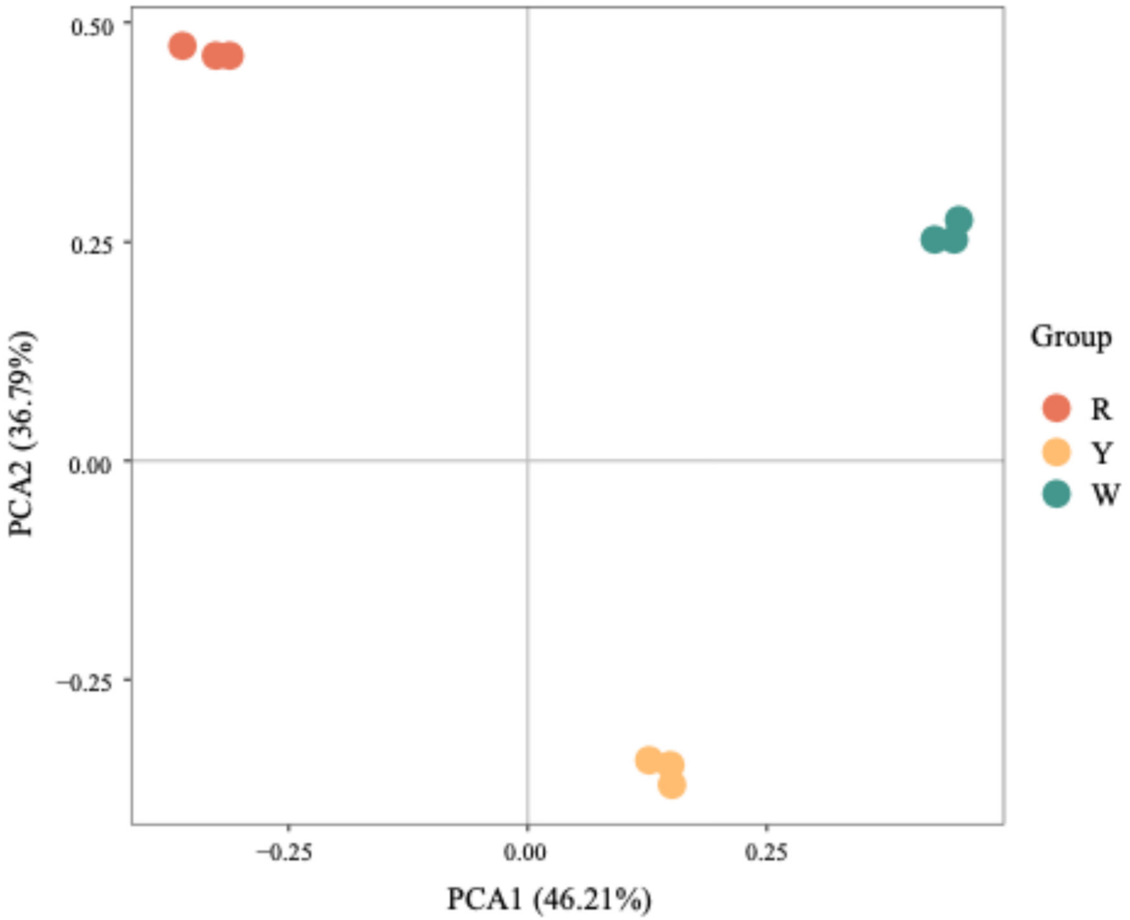

Figure S4. PCA score plots for all metabolites in three different colour groups

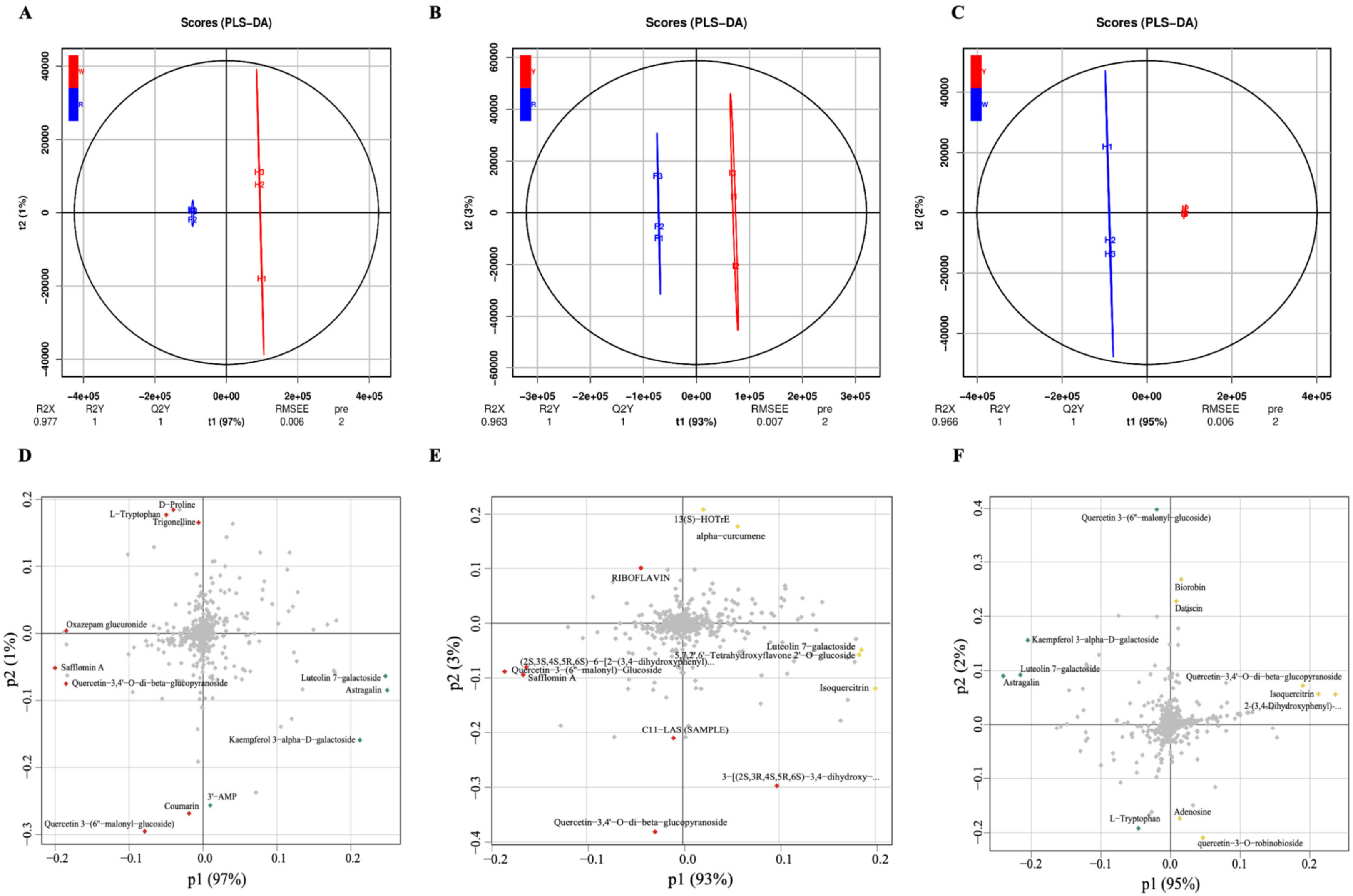
A
Scores (PLS−DA)
t2 (1%)
t1 (97%)
R2X
0.977
R2Y
1
Q2Y
1
RMSEE
0.006
pre
2
B
Scores (PLS−DA)
t2 (3%)
t1 (93%)
R2X
0.963
R2Y
1
Q2Y
1
RMSEE
0.007
pre
2
C
Scores (PLS−DA)
t2 (2%)
t1 (95%)
R2X
0.966
R2Y
1
Q2Y
1
RMSEE
0.006
pre
2
D
D−Proline
L−Tryptophan
Trigonelline
Oxazepam glucuronide
Safflomin A
Quercetin−3,4'−O−di−beta−glucopyranoside
Luteolin 7−galactoside
Astragalin
Kaempferol 3−alpha−D−galactoside
3'−AMP
Coumarin
Quercetin 3−(6"−malonyl−glucoside)
p2 (1%)
p1 (97%)
E
13(S)−HOTrE
alpha−curcumene
RIBOFLAVIN
Luteolin 7−galactoside
5,7,2',6'−Tetrahydroxyflavone 2'−O−glucoside
(2S,3S,4S,5R,6S)−6−[2−(3,4−dihydroxyphenyl)...
Quercetin−3−(6"−malonyl)−Glucoside
Safflomin A
Isoquercitrin
C11−LAS (SAMPLE)
3−[(2S,3R,4S,5R,6S)−3,4−dihydroxy−...
Quercetin−3,4'−O−di−beta−glucopyranoside
p2 (3%)
p1 (93%)
F
Quercetin 3−(6"−malonyl−glucoside)
Biorobin
Datiscin
Kaempferol 3−alpha−D−galactoside
Luteolin 7−galactoside
Astragalin
Quercetin−3,4'−O−di−beta−glucopyranoside
Isoquercitrin
2-(3,4-Dihydroxyphenyl)-...
Adenosine
L−Tryptophan
quercetin−3−O−robinobioside
p2 (2%)
p1 (95%)

Figure S5. The scores plot of OPLS-DA model discriminated the flower colours, with different colour groups. A: R vs. W OPLS-DA Analysis. B: R vs. Y OPLS-DA Analysis. C: W vs. Y OPLS-DA Analysis. D: Loading plot of R and W group DAMs. E: Loading plot of R and Y group DAMs. F: Loading plot of W and Y group DAMs.
