# Supplementary figures and images for: Integrated Genetic Diversity and Multi-Omics Analysis of Colour Formation in Safflower
Source: Int J Mol Sci. 2025 Jan 14;26(2):647. doi: 10.3390/ijms26020647 (PMC11765828; doi:10.3390/ijms26020647)

# Delta K

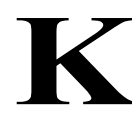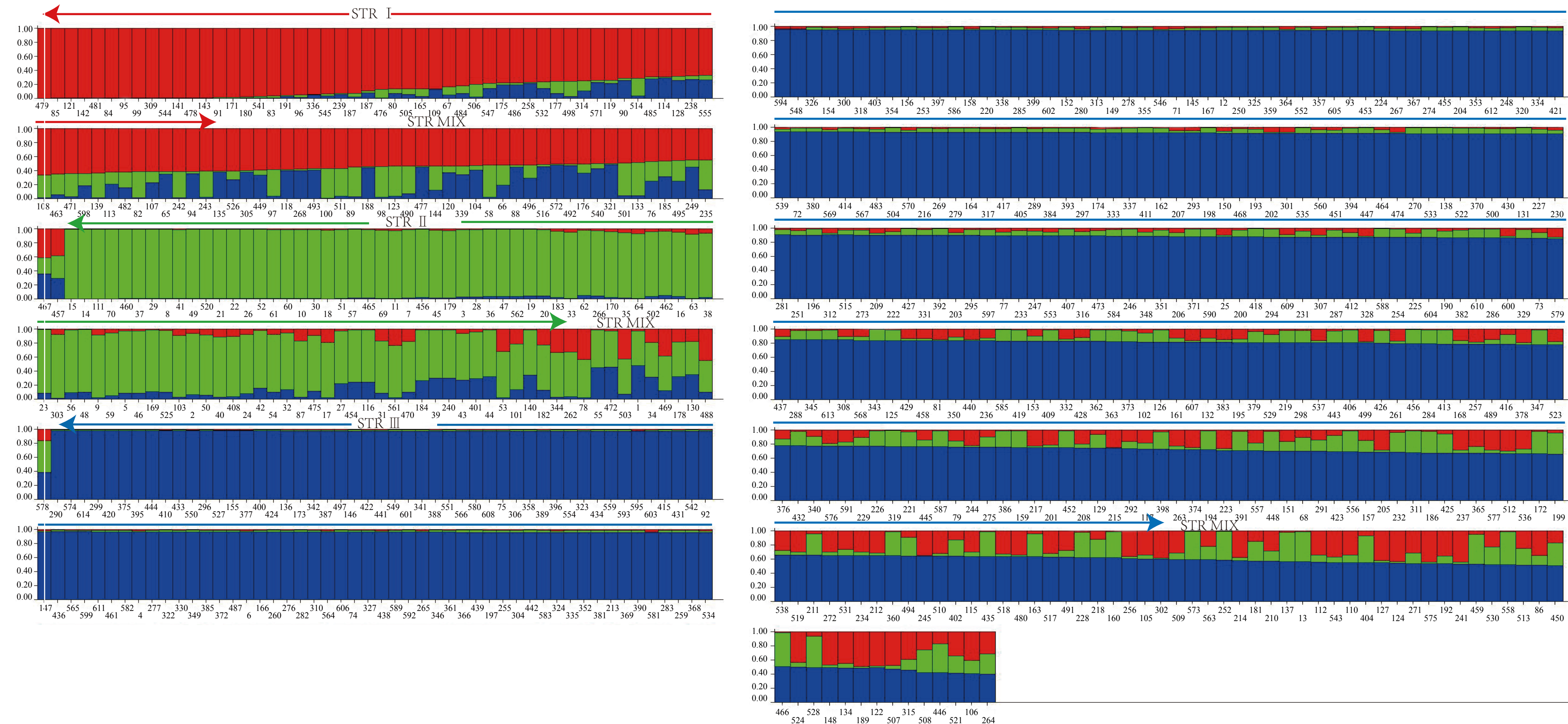

Supplement: Supplementary file 1 [file ijms-26-00647-s001.zip › FigS1.pdf]

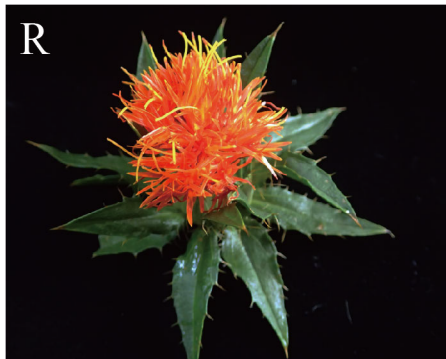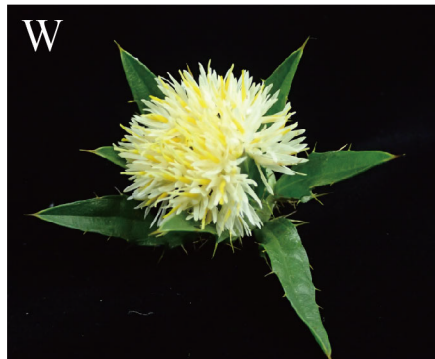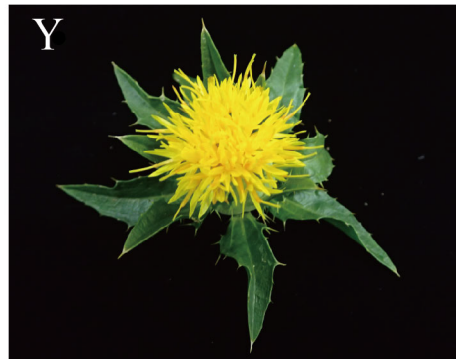

Supplement: Supplementary file 1 [file ijms-26-00647-s001.zip › FigS3.pdf]

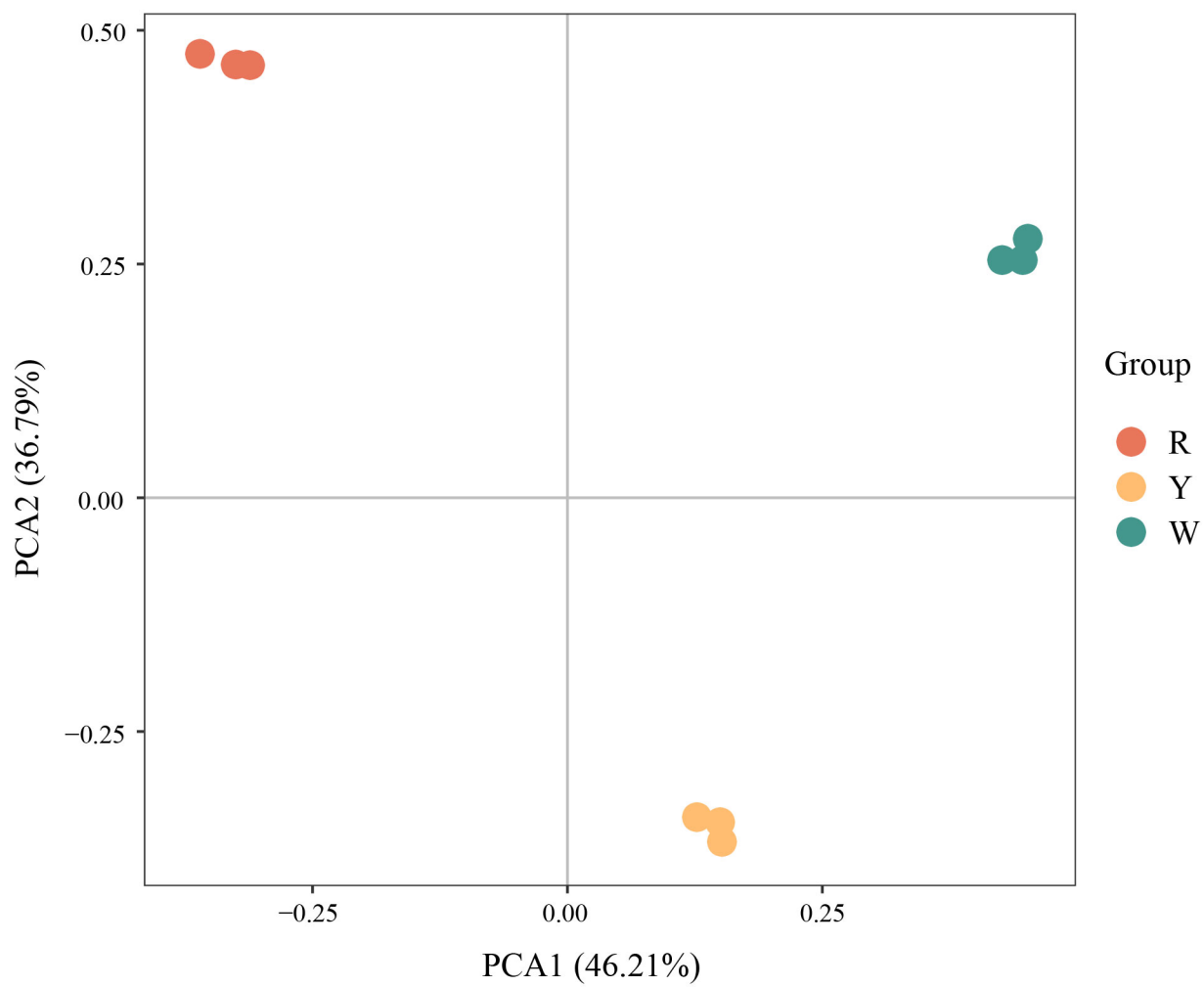

Supplement: Supplementary file 1 [file ijms-26-00647-s001.zip › FigS4.pdf]

A

Scores (PLS-DA)

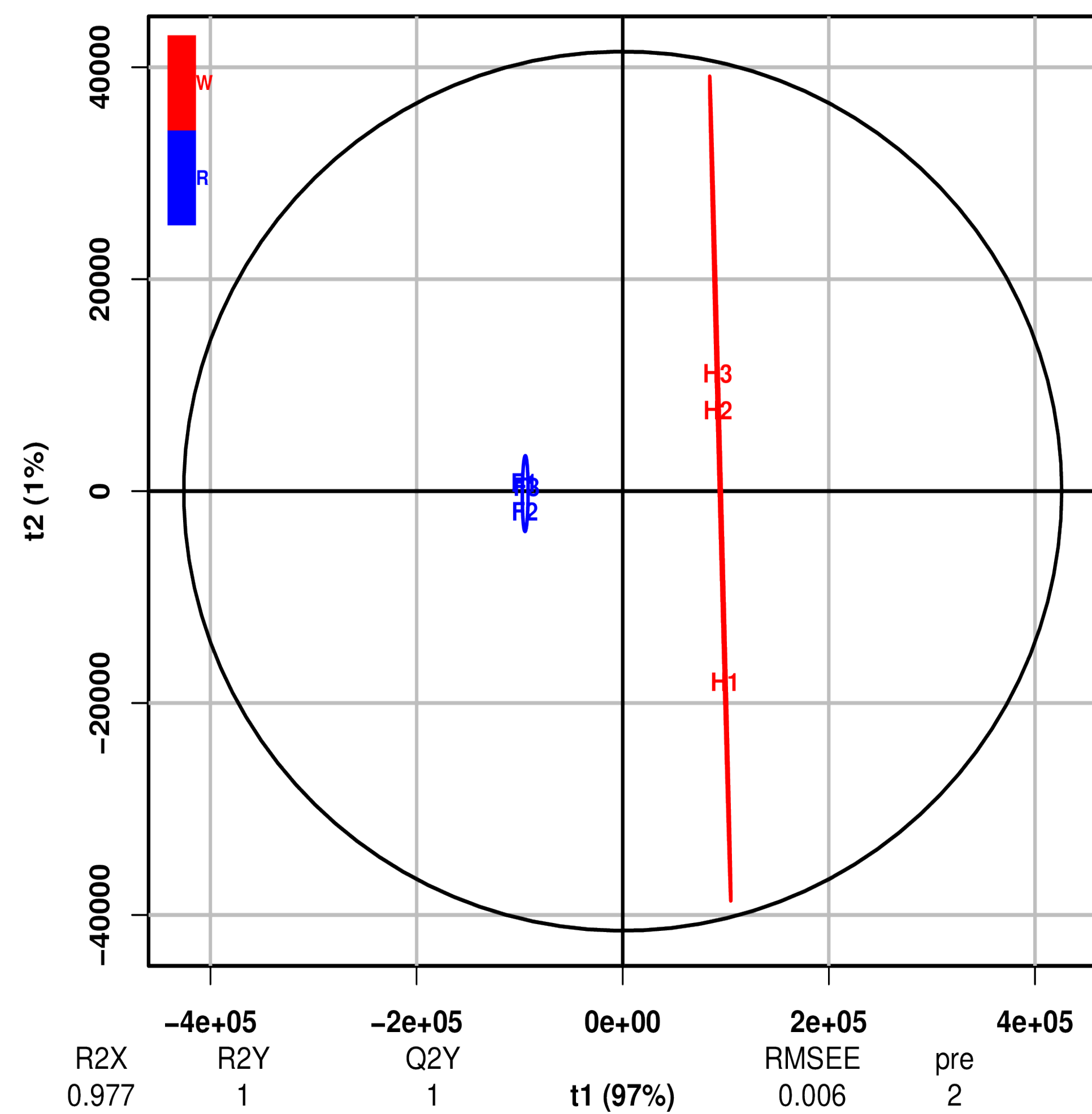

B

Scores (PLS-DA)

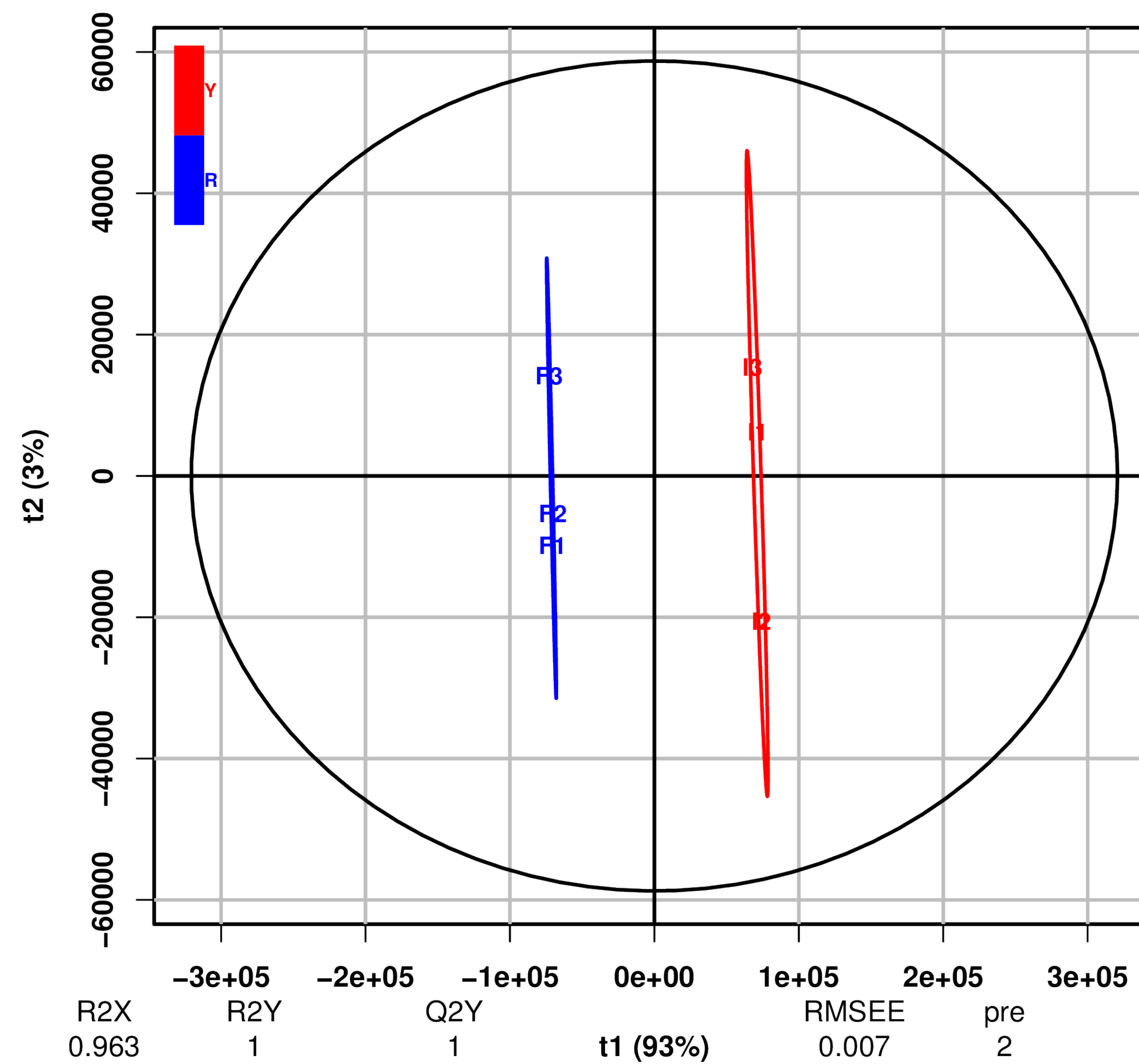

C

Scores (PLS-DA)

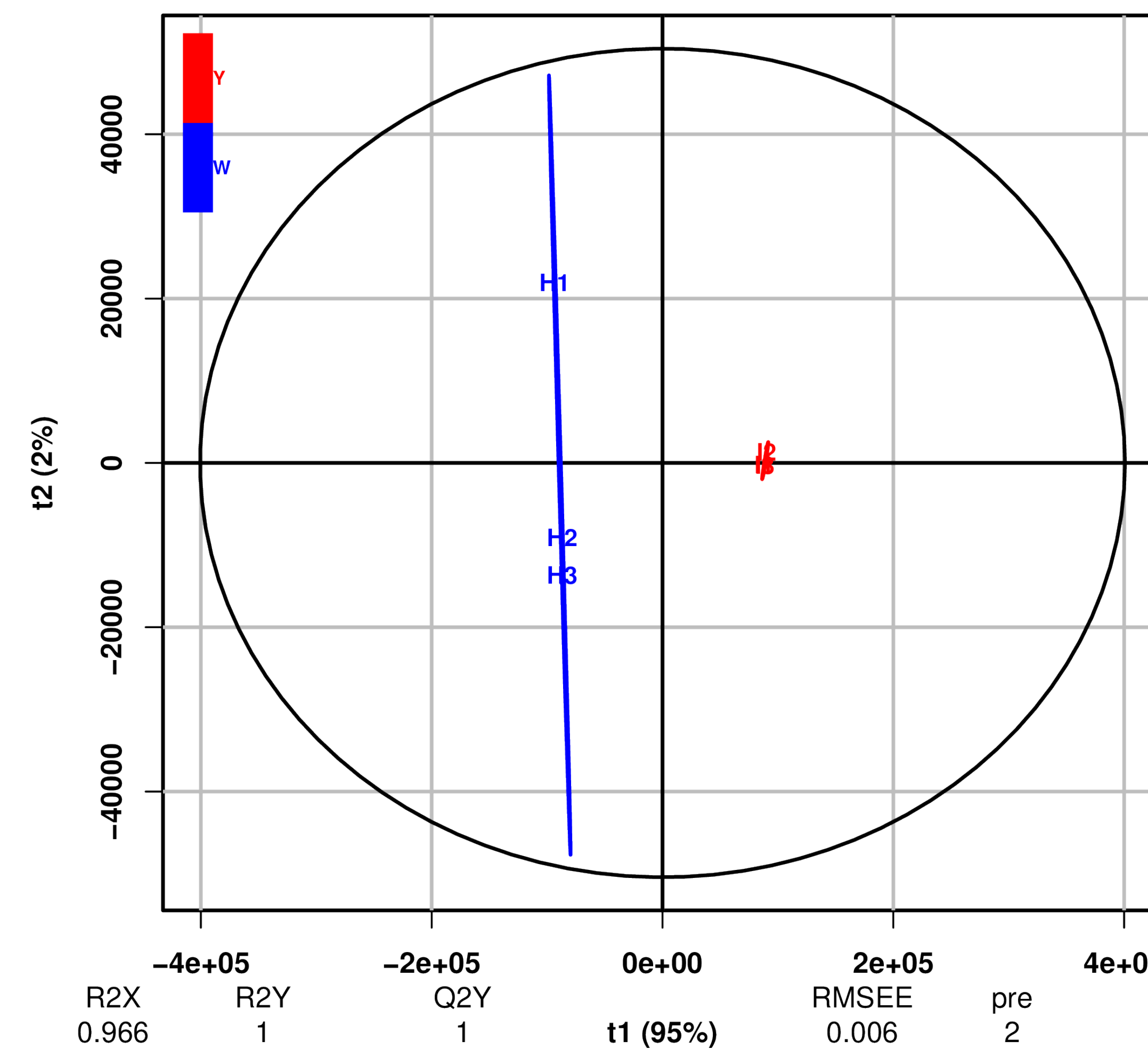

D

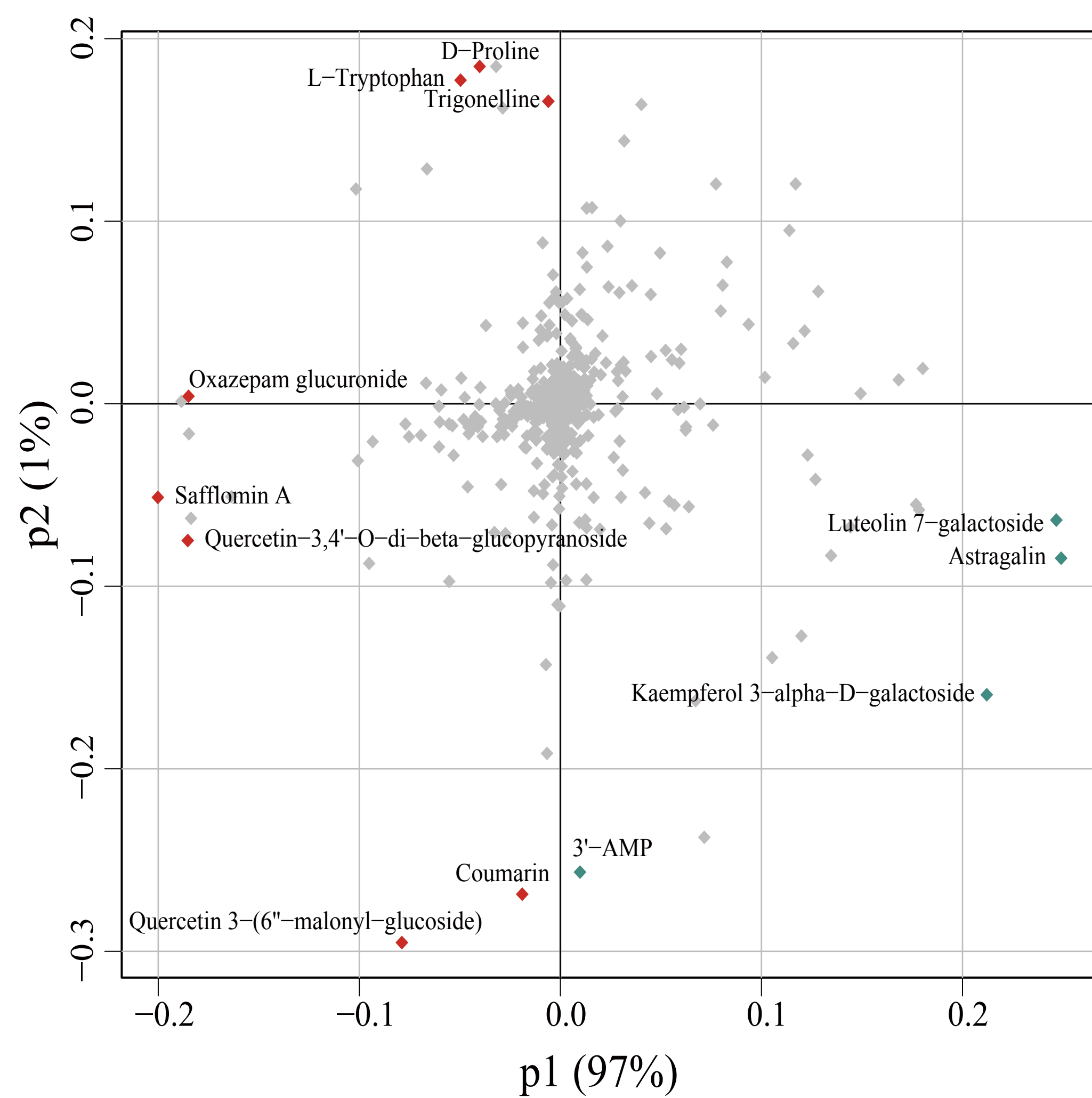

E

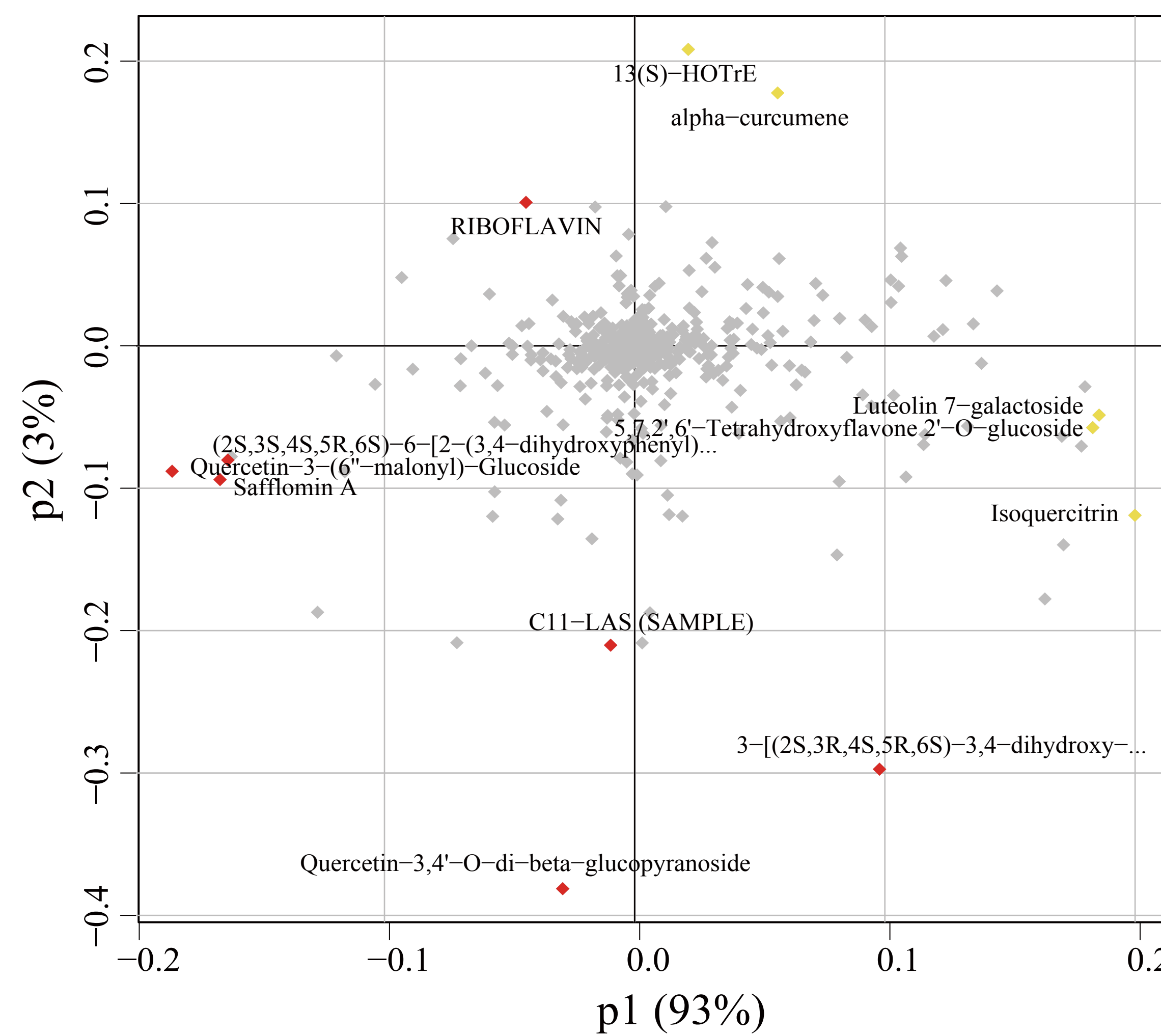

F

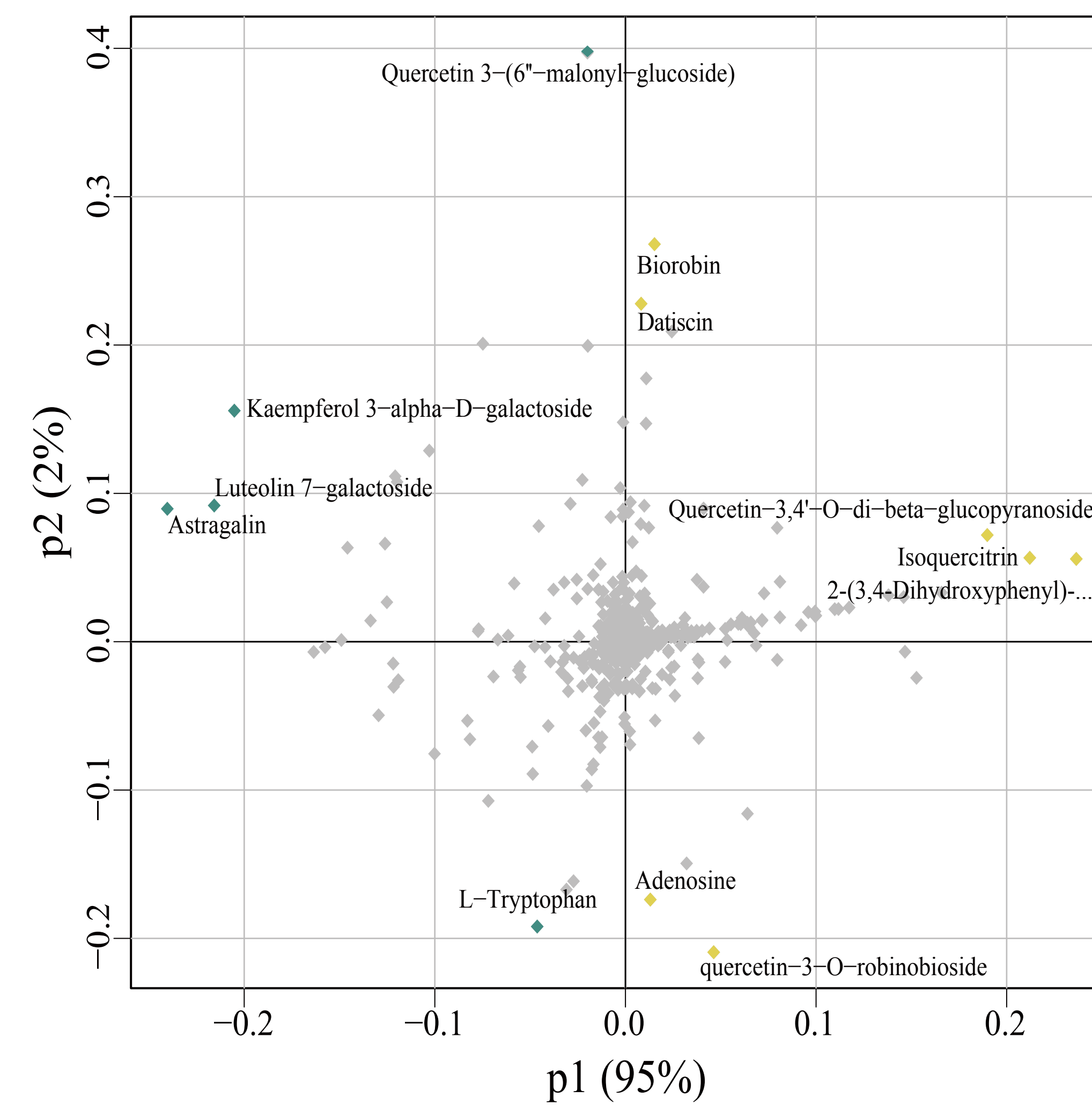

Supplement: Supplementary file 1 [file ijms-26-00647-s001.zip › FigS5.pdf]
